# Maternal and Paternal Caffeine Intake and ART Outcomes in Couples Referring to an Italian Fertility Clinic: A Prospective Cohort

**DOI:** 10.3390/nu10081116

**Published:** 2018-08-17

**Authors:** Elena Ricci, Stefania Noli, Sonia Cipriani, Irene La Vecchia, Francesca Chiaffarino, Stefania Ferrari, Paola Agnese Mauri, Marco Reschini, Luigi Fedele, Fabio Parazzini

**Affiliations:** 1 Dipartimento Madre-Bambino-Neonato, Fondazione IRCCS Ca’ Granda Ospedale Maggiore Policlinico, 20122 Milan, Italy; son.cipriani@gmail.com (S.C.); francesca.chiaffarino@gmail.com (F.C.); stefania.ferrari@policlinico.mi.it (S.F.); paola.mauri@unimi.it (P.A.M.); luigi.fedele@unimi.it (L.F.); fabio.parazzini@unimi.it (F.P.); 2 Department of Clinical Sciences and Community Health, Università di Milano, Fondazione IRCCS Ca’ Granda Ospedale Maggiore Policlinico, 20122 Milan, Italy; stefi.noli@gmail.com (S.N.); irene.lavecchia@unimi.it (I.L.V.); 3 Infertility Unit, Fondazione IRCCS Ca’ Granda Ospedale Maggiore Policlinico, 20122 Milan, Italy; m.reschini@gmail.com

**Keywords:** caffeine intake, assisted reproduction techniques, risk factors, implantation, clinical pregnancy, live birth

## Abstract

Caffeine intake, a frequent lifestyle exposure, has a number of biological effects. We designed a cohort study to investigate the relation between lifestyle and assisted reproduction technique (ART) outcomes. From September 2014 to December 2016, 339 subfertile couples referring to an Italian fertility clinic and eligible for ART procedures were enrolled in our study. Sociodemographic characteristics, smoking, and usual alcohol and caffeine consumption in the year prior to ART were recorded. The mean age of participants was 36.6 ± 3.6 years in women and 39.4 ± 5.2 years in men. After oocytes retrieval, 293 (86.4%) underwent implantation, 110 (32.4%) achieved clinical pregnancy, and 82 (24.2%) live birth. Maternal age was the main determinant of ART outcome. In a model including women’s age and college degree, smoking habits, calorie and alcohol intake for both partners, previous ART cycles, and partner’s caffeine intake, we did not observe any association between caffeine intake and ART outcome. Using the first tertile of caffeine intake by women as a reference, the adjusted rate ratio (ARR) for live birth was 1.09 (95% confidence interval (CI) 0.79–1.50) in the second and 0.99 (95% CI 0.71–1.40) in the third tertiles. In conclusion, a moderate caffeine intake by women and men in the year prior to the ART procedure was not associated with negative ART outcomes.

## 1. Introduction

### Caffeine Intake is Among the Most Common Lifestyle Exposure in Women and Men Alike 

Caffeine (1,3,7-trimethylxanthine) is found in coffee, tea, soft drinks (particularly cola-containing beverages and energy drinks), and chocolate. It easily crosses biologic membranes, is rapidly distributed throughout the body, and has been found in saliva, breast milk, the embryo, and the neonate [1]. The caffeine molecule is easily absorbed by humans, having approximately 100% bioavailability when taken by oral route and reaching a peak in the blood within 15–45 min after its consumption [2]. Caffeine has a number of biologic effects, including central nervous system stimulation, increased secretion of catecholamine, relaxation of smooth muscles, and stimulation of heart rate. Caffeine can also reach the follicular fluid, suggesting that it might exert a harmful role on the female reproductive process [3].

During the last decades, the relation between lifestyle factors and spontaneous fertility has been investigated in several observational studies, but, with regard to caffeine intake, few studies have analyzed the association between caffeine intake and in vitro fertilization (IVF) outcomes, showing inconsistent results. One study observed a negative association with live birth, when comparing women consuming >2–50 and >50 versus <2 mg/day of caffeine in the year prior to IVF [4], while other studies found no association between caffeine intake consumed just before or during IVF treatment and IVF outcomes [5,6]. In a study conducted in Boston [7], the adjusted percentage of cycles resulting in live birth for women in increasing categories of caffeine intake was 46% for <50 mg/day, 44%, 42%, 40% in intermediate intake categories, and 40% for >300 mg/day. On the other hand, Karmon et al. [8] recently found that caffeine intake was associated with a lower probability of achieving live birth after assisted reproduction techniques (ART), although this inverse association was limited to intracytoplasmic sperm injection (ICSI) cycles.

Thus, limited and conflicting data are available on the relation between caffeine intake and ART outcomes. In this paper, we analyzed the role of male and female caffeine consumption in ART outcomes, using data from a cohort study conducted in an Italian fertility center.

## 2. Methods

From September 2014 to December 2016, on randomly selected days, subfertile couples presenting for evaluation to the Fertility Unit of Fondazione IRCCS Ca’ Granda, Ospedale Maggiore, Policlinico, Milan, and eligible for assisted reproduction technologies (ART), were invited to participate in an ongoing prospective cohort study on the role of lifestyle habits and diet on ART outcome. The study protocol was approved by the Ethical Review Board of Fondazione IRCCS Ca’ Granda, Ospedale Maggiore, Policlinico (Milan, Italy). All procedures were conducted in accordance with the Helsinki Declaration and all participants provided written informed consent.

Study participation was proposed during the diagnostic phase. Couples were interviewed on the day of oocyte retrieval. On the same day, a semen sample was also collected and analyzed to proceed with in vitro fertilization (IVF) or intracytoplasmic sperm injection (ICSI). The time interval between the proposal of the study and the interview was generally less than one month. In the early period only women were interviewed, whereas partners’ information collection started at a later stage.

Both partners of couples who agreed to participate were interviewed by centrally trained personnel, using a standard questionnaire to obtain information on general sociodemographic characteristics, personal and health history and habits (including smoking, physical activity, alcohol intake, and methylxanthine-containing beverages consumption). Couples who did not speak fluent Italian were excluded.

The overall participation rate was close to 95%. This high participation rate was mainly due to the fact that couples were interviewed during the period spent waiting for the different diagnostic stages, before the actual ART procedure. Considering this down time and the not sensitive character questions, couples did not usually refuse to answer the questionnaire.

The questionnaire included information on sociodemographic characteristics, anthropometric variables, and lifestyle factors—including tobacco smoking, alcohol and caffeine intake, and diet habits—as well as a problem-oriented personal medical history and reproductive history.

Information on diet was based on a reproducible and validated food frequency questionnaire (FFQ), including 78 foods, food groups (such as the major sources of animal fats (i.e., red meat, milk, cheese, ham, salami), folates, vitamins (vegetables and fruit), pasta and bread consumption, cake, sweets and chocolate, fish), and the most common Italian recipes [9,10,11]. Patients were asked to report their usual weekly food consumption in the last year. The FFQ includes the average weekly consumption of 78 food items or food groups. Energy and mineral, macro-, and micronutrient intakes were estimated using the most recent update of an Italian food consumption database [12].

The weekly numbers of drinks for several alcoholic beverages were elicited from the subjects. Taking into account the different ethanol concentrations, one drink corresponded to approximately 125 mL of wine, 330 mL of beer, and 30 mL of hard liquor (i.e., about 12.5 g of ethanol). Total alcohol intake, expressed in grams of ethanol per day (g/day), was computed as the sum of all reported alcoholic beverages. “Never drinkers” were patients who abstained from drinking lifelong; “ex-drinkers” were individuals who had abstained from drinking for at least 12 months at the time of interview. For the purpose of this study, we considered these subjects in the category “abstainers”.

Further, questions included information on coffee and other methylxanthine-containing beverages (tea, cocoa, and decaffeinated coffee), and the average number of cups per day. Caffeine intake from coffee (60 mg per cup), cappuccino (75 mg per cup), tea (45 mg per cup), decaffeinated coffee (4 mg per cup), and chocolate (6 mg/10 g) was calculated [13].

A subject was considered a smoker if she had smoked more than one cigarette/day for at least one year; an ex-smoker if she had smoked more than one cigarette/day for at least one year, but had stopped more than one year before the interview, and a non-smoker if she had never smoked more than one cigarette/day.

Satisfactory reproducibility of questions on self-reported smoking and drinking habits in our study populations has been previously reported [14].

Patients were managed according to a standardized clinical protocol, as reported in detail elsewhere [15]. Couples underwent ART with conventional IVF or ICSI as clinically indicated.

Serum hCG assessment to detect pregnancy was performed 14 or 16 days after ovulation triggering or luteinizing hormone (LH) surge. Women with positive human corionic gonadotropin (hCG) values underwent a transvaginal sonography three weeks later. Clinical pregnancy was defined as the presence of at least one intrauterine gestational sac.

All clinical information (including infertility diagnoses) was collected from medical records. 

### Statistical Analysis

Multiple outcomes were considered in this analysis: (1) number of retrieved high quality oocytes; (2) undergoing embryo transfer (implantation); (3) clinical pregnancy; (4) live birth.

Categorical variables were described as frequency (N) and percentage (%) and compared using the Pearson or Mantel-Haenzsel (MH) chi-square, as appropriate. Continuous variables were described as means with standard deviation (SD) if normally distributed, or medians and interquartile ranges (IQR) if not normally distributed. Univariate analyses used were analysis of variance and Kruskal-Wallis test. The correlation between male and female caffeine consumption was evaluated by means of Spearman correlation rho, because caffeine consumption was not normally distributed.

In the multivariable models, we included as potential confounders variables associated with caffeine intake or ART outcomes at the univariate analysis. Thus, we accounted for women’s age, education, tobacco smoking, alcohol intake, total energy intake, and previous ART cycles. 

As regards the oocyte number, it was square-root transformed and included in a general linear equation with the aforementioned variables. We calculated the adjusted means in tertiles of women’s caffeine intake, and according to its 95% confidence intervals (CIs). Then, these figures were back-transformed to medians and 95% CIs. 

Using unconditional multiple logistic regression, we estimated rate ratios (RR) of each outcome and corresponding 95% CIs in categories of caffeine intake (approximate tertiles). In the logistic regression equation, we included woman’s age, education, tobacco smoking, alcohol intake, total energy intake, and previous ART cycles. As regards men’s variables, we included alcohol and calorie intake. Furthermore, we mutually adjusted men’s and women’s caffeine intake. In a second model, we combined categories of intake under and over the median (for each sex) and, using the lowest category (both the woman’s and partner’s intake under the median) as the reference, we calculated the RRs for ART failure in the other three groups.

Statistical significance was set at *p* < 0.05. All analyses were performed using SAS software, version 9.4 (SAS Institute, Inc., Cary, NC, USA).

## 3. Results

From September 2014 to December 2016, 501 women and 347 men were interviewed; since eight men did not provide complete information, the couples were excluded from the analysis. The final analysis included 339 couples, who provided complete information about their lifestyle and coffee/caffeine intake and underwent an ART cycle. 

As regards women, the mean age was 36.6 years (standard deviation, SD, 3.6, range 27–45) and the mean body mass index (BMI) was 22.2 kg/m^2^ (SD 3.7, range 17.0–41.0); 18 (5.4%) women were obese (BMI > 30.0 kg/m^2^). As regards men, the mean age was 39.4 years (SD 5.2, range 27–60) and the mean BMI was 25.3 kg/m^2^ (SD 3.0); 29 (8.8%) men were obese.

Table 1 shows the characteristics of women and men according to caffeine intake. There was no difference in terms of age, education, BMI, or cause for infertility in tertiles of caffeine consumption. A relationship was observed with smoking habits and alcohol intake both in men (chi-square *p* = 0.001 and 0.02, respectively) and women (chi-square *p* = 0.002 and 0.01, respectively). Women who had undergone previous ART cycles were more frequently in the lowest caffeine intake tertile (*p* = 0.002). Both men’s and women’s total energy intake increased by tertiles of caffeine consumption (*p* < 0.0001).

The correlation between male and female caffeine intake was statistically significant (*p* = 0.0002) but not very high (Spearman rho = 0.20).

After oocytes retrieval, 293 (86.4%) underwent embryo-transfer, 110 (32.4%) achieved clinical pregnancy, and 82 (24.2%) experienced a live birth, including eight twin births. Out of 28 interrupted clinical pregnancies, 27 were miscarriages and one was an induced abortion. 

ART outcomes were not associated with any men’s characteristics, whereas women’s education was significantly related to implantation (RR for college degree 1.78, 95% CI 1.00–3.18) and age at clinical pregnancy (for women aged: 35–40 years, RR 1.11, 95% CI 0.80–1.54; ≥40 years, RR 1.80, 95% CI 1.09–2.98; chi-square for trend 5.05, *p* = 0.025) and live birth (for women aged: 35–39 years, RR 1.30, 95% CI 0.88–1.93; ≥40, RR 2.43, 95% CI 1.28–4.63; chi-square for trend 7.93, *p* = 0.005). In both outcomes, older women were at a higher risk of failure. At univariate analysis, no association was observed with men’s or women’s caffeine intake.

Mean gestational week at delivery was 39.2 (SD 1.9, range 34–42); this was not associated with maternal caffeine intake, either as a continuous variable or in tertiles (Spearman rho = 0.19, *p* = 0.09). Mean gestational age in tertile of maternal intake was 39.0 (SD 1.7), 39.4 (SD 2.0), and 39.3 (SD 2.1) in the first, second, and third tertiles, respectively. Excluding twins, mean birth weight was 3140 (SD 428), with no significant differences across groups of maternal caffeine intake.

Table 2 shows the adjusted number of retrieved oocytes, adjusted for women’s age, education, smoking habits, and calorie and alcohol intake. Adjusted median number of oocytes was higher in the third tertile of caffeine intake, but this difference was not statistically significant.

RRs for caffeine intake, after adjusting for variables that were associated with caffeine intake (smoking habits and alcohol intake, daily calories) or ART outcomes (women’s age and education), was consistently higher in the intermediate class of women’s intake, but this findings were not significant. Men’s caffeine consumption was also not statistically significant; no dose-effect was suggested by the observed estimates. 

We built a second model with four categories for combined couple’s caffeine intake (lower and equal/higher than the median for their sex): using the group of lowest combined caffeine intake, we did not find significant associations between the outcomes and different couples’ caffeine intake, nor did we observe trends suggesting a relationship. 

Performing the analysis in groups of procedure (IVF or ICSI), we did not find any marked difference in the relationship between caffeine intake and ART outcomes (data not shown). 

## 4. Discussion

This prospective study of couples undergoing IVF or ICSI found that caffeine intake by women, men, and the couple was not associated with implantation, clinical pregnancy, and live birth, adjusting for women’s age class and college degree, smoking habits, calorie and alcohol intake for both men and women, and previous ART cycles. Similarly, caffeine consumption by women was not related to the number of oocytes retrieved.

Potential limitations of this study should be considered. All information on lifestyle habits was self-reported by the patients, so some underestimates may have occurred. However, in Italy, recommendations to avoid caffeine in pregnancy have not received widespread attention and are not routinely advocated by gynecologists before IVF or ICSI, and misreporting of this variable should be unlikely.

Other sources of bias, including selection or confounding factors, are also unlikely to have produced marked effects, especially considering that all subjects were interviewed in the same institution and that participation was practically complete.

With regard to other biases, we analyzed information on nutritional status, and their inclusion into the model did not change the estimated OR. Further, the questionnaire was satisfactorily reproducible: correlation coefficients were >0.65 for most frequently eaten food, and between 0.50 and 0.65 for others [16]. However, the exact amount of caffeine in caffeinated beverages is difficult to quantify. Although patients reported the number of cups of caffeinated beverages that they drink, the exact amount of milligrams of caffeine in a cup depends on the mix of the brew, how it is prepared, and the size of the cup. The questionnaire also asked questions about soda, but did not discriminate between caffeinated and non-caffeinated varieties. Although these factors are likely to underestimate the caffeine intake, a differential bias is unlikely.

Another potential limitation is study power. For example, with our data we can identify an RR of pregnancy loss for the third tertile of caffeine intake of about 1.8. Thus, our results cannot rule out modest effect sizes, which we were underpowered and thus difficult to detect.

The strengths of our study include its prospective design with complete follow-up and our ability to adjust for a wide range of potential confounders. We also obtained information on male partner diet, alcohol intake, and smoking habits, which previous studies have not included. 

We did not found any statistically significant association between caffeine intake and ART success.

Our findings are not consistent with those of Klonoff-Cohen et al. [4], who observed an association between usual coffee intake and lower ART success rate in 221 couples undergoing IVF or gamete intra-Fallopian transfer. This relationship was significant even in women reporting an intake of 20–50 mg caffeine/day, less than the equivalent of one cup of coffee per day. However, no association was observed with intake in the week before or during the procedure, a fact that the authors ascribed to the possibility that during the ART procedure women refrained from or decreased coffee drinking. 

In the study by Al-Saleh and colleagues [3], no relationship emerged between coffee consumption and pregnancy outcomes, yet the authors observed a decrease in the number of eggs retrieved and an increase in miscarriage frequency as caffeine intake increased.

On the contrary, a recent cohort study, including 300 women and 493 ART cycles, provided reassurance that low to moderate intakes of caffeine (e.g., <200 mg/day) in the year prior to infertility treatment initiation do not have an adverse effect on intermediate or clinical outcomes of ART [7]. Another recent study by Machtinger et al. [6] enrolled 340 women undergoing IVF from 2014 through 2016 and did not retrieve any association between coffee and caffeine consumption and ART outcomes, whereas a threat to reproductive success was attributable to sugared beverages, independent of their caffeine content.

Considering men’s caffeine intake as well, Choi et al. [5] found no relationship with implantation, fertilization, or live birth in a cohort including 2474 couples and 4716 IVF cycles. Although higher caffeine intake by women was associated with a significantly lower peak estradiol level, it was not related to the number of oocytes retrieved, implantation, fertilization, or live birth rate.

In a survey conducted in Italy in 2005–2006 [17], 1245 women had a median caffeine intake of 116 mg/day (95th percentile 355 mg/day) and 1068 men had a median caffeine intake of 112 mg/day (95th percentile 330 mg/day). In our sample, levels of consumption were similar in men, with a higher median (180 mg/day) but a similar 95th percentile, but not in women, who showed a similar median (126 mg/day) but a lower 95th percentile intake (272 mg/day). About 80% of women in our group consumed less than 200 mg of caffeine, which is the limit that, according to the European Food Safety Agency (EFSA), does not give rise to safety concerns for the fetus [18].

## 5. Conclusions

Our study does not show an effect of moderate coffee intake by women, men, or the couple on oocyte quality and success rate after ART procedures. Considering that our sample represented a moderate consumption of caffeine, as well as alcohol and tobacco, we cannot evaluate the effect of higher intakes on IVF outcomes. Thus, conservatively, all women seeking pregnancy should be advised to maintain caffeine intakes within limits suggested by the EFSA.

## Figures and Tables

**Table 1:** Demographic characteristics of 339 couples, according to caffeine intake.

	Women						Men					
	First Tertile		Second Tertile		Third Tertile		First Tertile		Second Tertile		Third Tertile	
	0–86 mg/day		87–180 mg/day		181–480 mg/day		0–124 mg/day		125–209 mg/day		210–560 mg/day	
	*n* = 114	33.6%	*n* = 116	34.2%	*n* = 109	32.1%	*n* = 112	33.1%	*n* = 113	33.3%	*n* = 114	33.6%
Daily Caffeine Intake (mg/day), Median (IQR)	37	14–60	128	111–147	215	188–255	62	17–100	180	154–189	258	231–310
**Age (years)**												
<35	31	27.2	33	28.4	36	33.0	19	17.0	25	22.1	25	21.9
35–39	60	52.6	51	44.0	52	47.7	39	34.8	43	38.0	42	36.8
≥40	23	20.2	32	27.6	21	19.3	54	48.2	45	39.8	47	41.2
**College Degree**	62	54.4	67	57.8	53	48.6	42	37.5	42	37.2	51	44.7
**Cause of Infertility**												
Unexplained	23	20.2	31	26.7	16	14.7	25	22.3	23	20.4	22	19.3
Female factor only	48	42.1	42	36.2	41	37.6	42	37.5	45	39.8	44	38.6
Male and female factor	43	37.7	43	37.1	52	47.7	45	40.2	45	39.8	48	42.1
**BMI**												
<18.5	12	10.5	4	3.5	12	11.0	-	-	-	-	-	-
18.5–24.9	82	71.9	86	76.1	78	71.6	46	45.1	55	48.7	45	39.5
25.0–29.9	17	14.9	13	11.5	14	12.8	48	47.1	48	42.5	58	50.9
≥30.0	3	2.6	10	8.8	5	4.6	8	7.8	10	8.8	11	9.6
**Smoking Habits**												
Never	**74**	**64.9**	**72**	**62.1**	**49**	**45.0**	**55**	**53.4**	**46**	**40.7**	**28**	**24.8**
Current	**15**	**13.2**	**15**	**12.9**	**34**	**31.2**	**24**	**23.3**	**33**	**29.2**	**50**	**44.2**
Former	**25**	**21.9**	**29**	**25.0**	**26**	**23.8**	**24**	**23.3**	**34**	**30.1**	**35**	**31.0**
**Alcohol Intake**												
Abstainer	**40**	**35.1**	**33**	**28.4**	**23**	**21.1**	**14**	**13.6**	**9**	**8.0**	**8**	**7.0**
<1 unit/day	**72**	**63.2**	**75**	**64.7**	**73**	**67.0**	**57**	**55.3**	**66**	**58.4**	**53**	**46.5**
≥1 unit/day	**2**	**1.7**	**8**	**6.9**	**13**	**11.9**	**32**	**31.1**	**38**	**33.6**	**53**	**46.5**
**Leisure Physical Activity**												
<2 h/week	57	50.0	66	57.4	69	63.3	36	35.3	48	42.9	49	43.8
2–4	44	38.6	43	37.4	32	29.4	37	36.3	44	39.3	38	33.9
>4	13	11.4	6	5.2	8	7.3	29	28.4	20	17.9	25	22.3
**Previous ART Cycle**	**81**	**71.0**	**60**	**51.7**	**54**	**49.5**	67	59.8	63	58.4	62	54.4
**Daily Calories Intake** **(Kcal/day), Median (IQR)**	**1589**	**1367–1924**	**1748**	**1497–2124**	**1871**	**1602–2179**	**1781**	**1480-2060**	**1960**	**1649–2262**	**2122**	**1753–2407**

Sometimes the sums do not add up to the totals because of missing values. Bold: *p* < 0.05, IQR: median and interquartile range; BMI: body mass index.

**Table 2:** Rate ratios for failure of assisted reproduction technique (ART) outcomes.

	Number of High-Quality Oocytes (Adjusted * Median, 95% CI)	Implantation				ARR (95% CI)	Clinical Pregnancy				ARR (95% CI)	Live Birth				ARR (95% CI)
		Failure *n* = 46 (13.6%)		Success *n* = 293 (86.4%)			Failure *n* = 229 (67.6%)		Success *n* = 110 (32.4%)			Failure *n* = 257 (75.8%)		Success *n* = 82 (24.2%)		
**Women**																
**Caffeine intake**		***n***	**%**	***n***	**%**		***n***	**%**	***n***	**%**		***n***	**%**	***n***	**%**	
First tertile	4.8 (4.0–5.7)	14	30.4	100	34.1	1	74	32.3	40	36.4	1	84	32.7	30	36.6	1
Second tertile	4.6 (3.9–5.4)	20	43.5	96	32.8	1.34 (0.64–2.79)	84	36.7	32	29.1	1.07 (0.76–1.50)	95	37.0	21	25.6	1.09 (0.79–1.50)
Third tertile	5.3 (4.5–6.1)	12	26.1	97	33.1	0.90 (0.38–2.10)	71	31.0	38	34.6	1.00 (0.70–1.43)	78	30.3	31	37.8	0.99 (0.71–1.40)
>90 percentile ^§^	5.1 (3.8–6.7)	5	10.9	29	9.9	0.58 (0.16–2.04)	23	10.0	11	10.0	0.96 (0.51–1.80)	24	9.3	10	12.2	0.99 (0.54–1.84)
**Men**																
**Caffeine intake**																
First tertile	-	12	26.1	100	34.1	1	71	31.0	41	37.3	1	83	32.3	29	35.4	1
Second tertile	-	22	47.8	91	31.1	1.64 (0.78–3.44)	76	33.2	37	33.6	1.01 (0.72–1.42)	88	34.2	25	30.5	1.02 (0.75–1.41)
Third tertile	-	12	34.8	102	34.8	0.78 (0.32–1.87)	82	35.8	32	29.1	1.07 (0.76–1.52)	86	33.5	28	34.1	1.00 (0.72–1.40)
>90 percentile ^§^	-	4	8.7	35	12	0.73 (0.17–3.16)	27	11.8	12	10.9	1.12 (0.59–2.14)	29	11.3	10	12.2	0.98 (0.54–1.83)
**Combined intake (W-M)**																
Low-low	-	14	30.4	81	27.6	1	64	28.0	31	28.2	1	71	27.6	24	29.3	1
Low-high	-	10	21.7	65	22.2	0.79 (0.34–1.85)	56	24.4	19	17.3	1.03 (0.71–1.50)	62	24.1	13	15.8	1.09 (0.76–1.55)
High-low	-	12	26.1	61	20.8	1.22 (0.53–2.78)	48	21.0	25	22.7	0.95 (0.64–1.42)	57	22.2	16	19.5	1.06 (0.73–1.54)
High-high	-	10	21.7	86	29.4	0.60 (0.24–1.48)	61	26.6	35	31.8	0.89 (0.61–1.30)	67	26.1	29	35.4	0.93 (0.65–1.33)

The final model included women’s age class and college degree, smoking habits, calorie and alcohol intake for both men and women, previous ART cycles, and partner’s caffeine intake. ARR: adjusted rate ratio; CI: confidence interval; * for women’s age class and education, smoking habits, calorie and alcohol intake; ^§^ reference category: <10 percentile; W-M: women-men.
